# Performance Characterization of a Hybrid Satellite-Terrestrial System with Co-Channel Interference over Generalized Fading Channels

**DOI:** 10.3390/s16081236

**Published:** 2016-08-05

**Authors:** Umer Javed, Di He, Peilin Liu

**Affiliations:** Shanghai Key Laboratory of Navigation and Location-Based Services, Shanghai Jiao Tong University, 800 Dongchuan Road, Shanghai 200240, China; dihe@sjtu.edu.cn (D.H.); liupeilin@sjtu.edu.cn (P.L.)

**Keywords:** hybrid satellite-terrestrial system (HSTS), amplify-and-forward (AF) relay, co-channel interference (CCI), land mobile satellite (LMS) channel, M-PSK, M-QAM, M-PAM

## Abstract

The transmission of signals in a hybrid satellite-terrestrial system (HSTS) in the presence of co-channel interference (CCI) is considered in this study. Specifically, we examine the problem of amplify-and-forward (AF)-based relaying in a hybrid satellite-terrestrial link, where the relay node is operating in the presence of a dominant co-channel interferer. It is assumed that direct connection between a source node (satellite) and a destination node (terrestrial receiver) is not available due to masking by obstacles in the surrounding. The destination node is only able to receive signals from the satellite with the help of a relay node located at the ground. In the proposed HSTS, the satellite-relay channel follows the shadowed Rice fading; and the channels of interferer-relay and relay-destination links experience generalized Nakagami-*m* fading. For the considered AF-based HSTS, we first develop the analytical expression for the moment generating function (MGF) of the overall output signal-to-interference-plus-noise ratio (SINR). Then, based on the derived exact MGF, we derive novel expressions for the average symbol error rate (SER) of the considered HSTS for the following digital modulation techniques: M-ary phase shift keying (M-PSK), M-ary quadrature amplitude modulation (M-QAM) and M-ary pulse amplitude modulation (M-PAM). To significantly reduce the computational complexity for utility in system-level simulations, simple analytical approximation for the exact SER in the high signal-to-noise ratio (SNR) regime is presented to provide key insights. Finally, numerical results and the corresponding analysis are presented to demonstrate the effectiveness of the developed performance evaluation framework and to view the impact of CCI on the considered HSTS under varying channel conditions and with different modulation schemes.

## 1. Introduction

The use of satellite communication systems nowadays is widespread in many diverse applications, such as navigation, mobile communication, broadcasting and disaster relief. Therefore, their proper working in the above diverse practical scenarios is very important for both the users and service providers. One highly probable event in land mobile satellite communications is the difficulty in maintaining line-of-sight (LOS) communications [1,2], because of the following propagation impairments: the blocking of signals caused by large obstacles (shadowing), severe attenuation (path loss) and the multipath channel exhibiting frequency selective fading may cause intersymbol interference [3,4]. This situation is also commonly called masking when LOS is no longer available between satellite and terrestrial users, and it severely affects the indoor users or in the case of low satellite elevation angles.

A number of works have studied the performance of hybrid satellite-terrestrial system (HSTS) [1,2] in the presence of the masking effect, e.g., [5,6,7,8,9,10]. The average symbol error rate (SER) of the fixed gain amplify-and-forward (AF) hybrid satellite terrestrial-system with generalized fading channels was derived in [6]. The analysis of cooperative relaying strategies combined with the delay diversity technique in a digital video broadcasting-satellite services to handhelds (DVB-SH)-compliant hybrid satellite/terrestrial network, for reducing the impairments caused by the masking, was given in [7]. A cooperative diversity scheme for mobile satellite multimedia broadcasting systems, utilizing space-time block codes and rate-compatible turbo codes to achieve diversity gains and additional coding gains, respectively, was analyzed in [8]. A hybrid satellite-terrestrial networking approach, where land mobile users have both satellite communication and cooperative-networking capabilities, to solve the masking problem was proposed in [9]. The use of different cooperative transmission schemes for the delivery of satellite services, in which mobile terminals forward the received signal from the satellite, was studied in [10]. The outage performance of an HSTS was analytically evaluated in [11]. The performance of a selective decode-and-forward (DF) protocol-based HSTS, employing the selection of the best relay terminal, was investigated in terms of outage probability by [12]. The symbol error probability of the hybrid/integrated satellite-terrestrial cooperative network described in [12] was evaluated in [13]. In [14], the authors considered the performance of AF-based relaying in a hybrid satellite-terrestrial free space optical (FSO) cooperative link, where a masked destination node was made to receive the relayed signals through an FSO link. The use of multiple antenna techniques in HSTS with masked ground node was considered in [15].

All of the above cited papers have contributed to our understanding of the performance analysis of HSTS; however, they have focused on the ideal case without co-channel interference (CCI). The assumption of no CCI is impractical nowadays due to the deployment of many wireless standards in the same spatial location and the increased practice of reusing the spectrum resource in traditional wireless networks [16,17,18]. The effect of CCI on the average SER of a particular HSTS was investigated in [19], where the DF protocol was used at the terrestrial relay, and the destination was corrupted by multiple Rayleigh faded interferers. The LOS path between satellite and destination node was assumed to be available in [19].

As is evident from the above literature survey, with the exception of [19], the effect of interference on the performance characteristics of dual-hop HSTS has not been investigated before in the open literature. However, we notice that the work in [19] was limited in the sense of practical deployment, due to the following reasons: DF was used at the relay instead of more general AF; a direct path between the satellite and destination was present (which is uncommon in urban canyons and indoor environments); and Rayleigh fading was used instead of generalized Nakagami-*m* fading. A more recent and practically oriented study was conducted in [20], where exact performance analysis of AF-based HSTS with co-channel interference was presented, considering multiple independent interferers and the effects of different channel parameters in a network. However, we note that the cumbersome performance analysis framework of [20] can be simplified with reasonable system model simplifications without any loss in practical insight, e.g., by considering single dominant co-channel interferer. Furthermore, extensions to other digital modulation techniques are possible in addition to M-ary phase shift keying (M-PSK) studied therein, such as commonly-used M-ary pulse amplitude modulation (M-PAM) and M-ary quadrature amplitude modulation (M-QAM). For M-QAM with chunk-based resource allocation in orthogonal frequency-division multiple access (OFDMA) systems, the papers [3,4] have given its performance in Rayleigh fading. Furthermore, the mathematical expressions for a simple, yet accurate high signal-to-noise ratio (SNR) approximation (asymptotic SER) of the exact SER, not provided in [20], could also be provided. As multiple co-channel-independent interferers were considered in [20], therefore, the resulting performance analysis developed was too cumbersome and tedious for the purpose of system-level analysis and simulations.

Specifically, we point out that computationally-simple and holistic performance characterization of three node AF-based HSTS for generalized fading channels, with noise and CCI at the relay, is not reported in the open literature despite the presence of some loosely-related studies. Therefore, in this work, we deal with the many open problems/issues (explained in the last paragraph) regarding the performance analyses of HSTS and develop the holistic and computationally-tractable performance analysis framework, which also addresses all of the shortcomings in [19,20] explained previously. As a result, the novelty and main contributions of this work for HSTS can be listed as follows: (1) We analyze the average SER of a digitally-modulated dual-hop fixed gain AF-based relay network with interference and noise at the relay, while the destination only experiences noise. We derive the average SER for the following constellations: M-PSK, M-QAM and M-PAM; (2) We consider a network where a direct connection between source node (satellite) and destination node (terrestrial receiver) is absent, so a terrestrial relay forwards the source symbol to the destination; (3) We assume generalized fading channels where the source-relay link follows the shadowed Rice LMS model [21]; and the channels of the relay-destination and interferer-relay links follow the Nakagami-*m* model; (4) Using the moment generating function (MGF)-based approach [22], the exact MGF of the proposed HSTS is derived, based on the derived MGF, the average SER of the considered network is given; (5) Finally, and importantly, we develop a simple (in terms of computational complexity), yet appropriately accurate high SNR approximation (asymptotic SER) for the exact SER, which provides important insight in the high SNR regime. Extensive numerical results in terms of the average SER and the asymptotic SER of the considered system model are provided in this paper. According to the authors’ best knowledge, this practically significant performance characterization of HSTS with CCI over generalized fading channels, which is analytically holistic while being of low computational complexity, shows the novelty of the presented work. Furthermore, for the considered HSTS, all of the mathematical expressions derived and the methods used, as well as the corresponding analyses presented here are reported in the literature for the first time and, so, show the novelty of this study.

The remainder of this article is organized as follows. Section 2 gives the detailed description of the considered dual-hop relay network with co-channel interference. Section 3 develops the performance analysis framework of the proposed system model. Section 4 derives the computationally-efficient asymptotic average SER. Section 5 presents the detailed numerical results. Finally, Section 6 concludes this paper.

## 2. System Model

**Figure 1:**
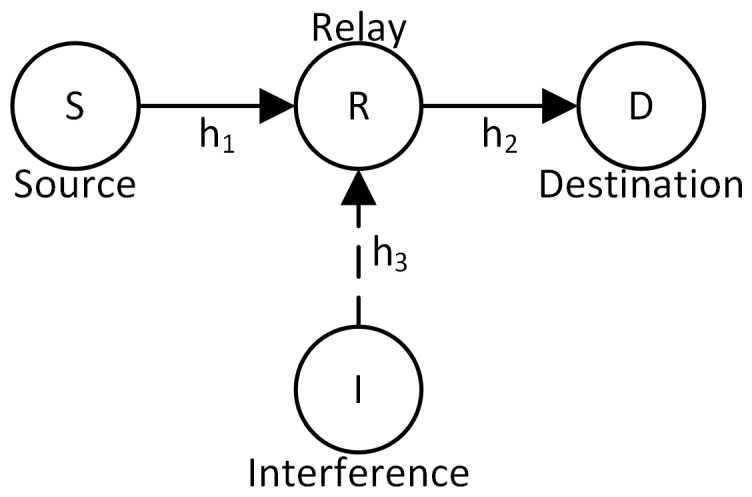
A hybrid/integrated satellite-terrestrial system with co-channel interference (CCI).

We consider an HSTS, where a satellite communicates with a destination node at the ground with the help of a relay node located at the ground, as shown in Figure 1. Therefore, there is no direct connection between the satellite and destination node. The overall communication is divided into two orthogonal phases. In the first phase, the satellite transmits its signal to the relay. At the relay, the received signal in the presence of a single dominant interferer will be:

(1) $$y_{1}=h_{1}x+h_{3}y+n_{1}$$

where $h_{1}$ is the channel gain between the satellite and the relay; *x* is the satellite’s transmitted symbol with $E_{s}$ power; $h_{3}$ is the channel gain between the interferer and the relay; *y* is the interferer’s transmitted symbol with $E_{i}$ power; $n_{1}$ is the zero-mean additive white Gaussian noise (AWGN) at the relay with $\sigma_{1}^{2}$ variance.

In the second phase, the relay multiplies the received signal $y_{1}$ with a fixed gain G>0 and forwards the amplified signal to the destination. The received signal at the destination, by using Equation (1), is given by:

(2) $$y_{2}=Gh_{2}\left(h_{1}x+h_{3}y+n_{1}\right)+n_{2}$$

where $h_{2}$ is the channel gain between the relay and the destination and $n_{2}$ is the zero-mean AWGN at the destination with $\sigma_{2}^{2}$ variance.

The satellite-relay link is assumed to follow the shadowed Rice fading channel with the following probability density function (PDF) [21]:

(3) $$f_{|h_{1}{|}^{2}}\left(x\right)=\alpha_{1}e^{-\beta_{1}x}{}_{1}F_{1}\left(m_{1};1;\delta_{1}x\right),\left(x\geq 0\right)$$

where $\alpha_{1}=\frac{1}{2b_{1}}{\left(\frac{2b_{1}m_{1}}{2b_{1}m_{1}+\Omega_{1}}\right)}^{m_{1}}$, $\delta_{1}=\frac{\Omega_{1}}{2b_{1}\left(2b_{1}m_{1}+\Omega_{1}\right)}$, $\beta_{1}=\frac{1}{2b_{1}}$ $\Omega_{1}$ is the average power of the LOS component, $2b_{1}$ is the average power of the multipath component, ${}_{1}F_{1}\left(a;b;z\right)$ is the confluent hypergeometric function [23] and $0\leq m_{1}\leq \infty$ is the Nakagami parameter. The relay-destination channel is assumed to follow the Nakagami-*m* distribution; hence, $|h_{2}{|}^{2}$ follows the Gamma distribution [6] as:

(4) $$f_{|h_{2}{|}^{2}}\left(x\right)=\lambda_{2}x^{m_{2}-1}e^{-\epsilon_{2}x},\left(x\geq 0\right)$$

where $\lambda_{2}=\frac{m_{2}^{m_{2}}}{\Omega_{2}^{m_{2}}\Gamma \left(m_{2}\right)}$, $\epsilon_{2}=\frac{m_{2}}{\Omega_{2}}$; and $\frac{1}{2}\leq m_{2}\leq \infty$ and $\Omega_{2}$ denote the shape and scale parameters, respectively, of the relay-destination channel. The interferer-relay channel is also assumed to follow the Nakagami-*m* distribution; hence, $|h_{3}{|}^{2}$ follows the Gamma distribution [6] as:

(5) $$f_{|h_{3}{|}^{2}}\left(x\right)=\lambda_{3}x^{m_{3}-1}e^{-\epsilon_{3}x},\left(x\geq 0\right)$$

where $\lambda_{3}=\frac{m_{3}^{m_{3}}}{\Omega_{3}^{m_{3}}\Gamma \left(m_{3}\right)}$, $\epsilon_{3}=\frac{m_{3}}{\Omega_{3}}$; and $\frac{1}{2}\leq m_{3}\leq \infty$ and $\Omega_{3}$ denote the shape and scale parameters, respectively, of the interferer-relay channel.

Here, we comment on the choice of the selected channel models for different links, i.e., the shadowed Rice and the Nakagami-*m*. The satellite-relay link (satellite-to-terrestrial wireless communication channel) is assumed to follow the shadowed Rice model, which is an example of the LMS channel, while the relay-destination link (terrestrial wireless communication channel) and interferer-relay link (terrestrial wireless communication channel) are assumed to follow the Nakagami-*m* model. The satellite-to-terrestrial propagation channels are inherently different from the terrestrial propagation channels because of the differences in the following underlying properties of the wireless channels: multipath fading (diffraction, reflection and scattering of the transmitted signal), LOS obstruction, path loss and shadowing. The above listed factors affect the signal level, while the channel additionally causes temporal dispersion and the Doppler shifts. The resulting first- and second-order statistics of the models are different due to these two different propagation environments. Therefore, we have selected the most widely-used channel models in their respective categories, i.e., Nakagami-*m* for the traditional wireless channel and shadowed Rice for the LMS channel. The evaluation of the distribution of the sum of squared shadowed-Rice random variables and its application to the LMS channel was carried out in [24]. The performance evaluation of an energy detector under multi-path fading/shadowing effects in a Gamma-shadowed Rician fading condition was done in [25].

Thus, we can see that the HSTS model under consideration alleviates the need of statistics for the sum of squared Nakagami-*m* random variables, required for multiple independent interferers [20]. As previously published results are either in the form of infinite sums or higher order derivatives of the fading parameter *m*, this makes the resulting modeling and analysis impractical and quite difficult to realize computationally [20]. Our work captures the essence of HSTS in [20] with the dominant single interferer, reduces the resulting approximations assumed therein and extends it to multiple dimensions.

## 3. Performance Analysis Framework

In the following, we will derive the average SER of our proposed system model. We will follow the standard MGF-based approach [22]. The overall signal-to-interference-plus-noise ratio (SINR) *γ* can be obtained [26], by using Equation (2), as:

(6) $$\gamma =\frac{E_{s}|h_{1}{|}^{2}{\left|h_{2}\right|}^{2}}{|h_{2}{|}^{2}\sigma_{1}^{2}+E_{i}|h_{2}{|}^{2}{\left|h_{3}\right|}^{2}+\frac{\sigma_{2}^{2}}{G^{2}}}$$

Alternatively, Equation (6) can be written as:

(7) $$\gamma =\frac{|h_{1}{|}^{2}{\left|h_{2}\right|}^{2}}{|h_{2}{|}^{2}+\frac{E_{i}}{\sigma_{1}^{2}}|h_{2}{|}^{2}{\left|h_{3}\right|}^{2}+\frac{\sigma_{2}^{2}}{\sigma_{1}^{2}G^{2}}}\frac{E_{s}}{\sigma_{1}^{2}}$$

On substituting $C=\frac{\sigma_{2}^{2}}{\sigma_{1}^{2}G^{2}}$ in Equation (7), we finally obtain:

(8) $$\begin{array}{cc} \gamma & =\frac{|h_{1}{|}^{2}{\left|h_{2}\right|}^{2}}{|h_{2}{|}^{2}+\frac{E_{i}}{\sigma_{1}^{2}}|h_{2}{|}^{2}{\left|h_{3}\right|}^{2}+C}\frac{E_{s}}{\sigma_{1}^{2}}\;\\ & =\frac{|h_{1}{|}^{2}{\left|h_{2}\right|}^{2}}{|h_{2}{|}^{2}+{\bar{\gamma }}_{int}|h_{2}{|}^{2}{\left|h_{3}\right|}^{2}+C}\bar{\gamma } \end{array}$$

where $\bar{\gamma }={\bar{\gamma }}_{1}=\frac{E_{s}}{\sigma_{1}^{2}}$, and ${\bar{\gamma }}_{int}={\bar{\gamma }}_{3}=\frac{E_{i}}{\sigma_{1}^{2}}$ are the per hop average signal-to-noise-ratio (SNR) and interference-to-noise-ratio (INR), respectively.

### 3.1. Calculation of the MGF of the HSTS

The MGF of the considered system model can be written, by using Equation (8), as:

(9) $$\begin{array}{cc} M_{\gamma }\left(s\right) & =E_{\gamma }\left[e^{-s\frac{|h_{1}{|}^{2}{\left|h_{2}\right|}^{2}}{|h_{2}{|}^{2}+\frac{E_{i}}{\sigma_{1}^{2}}|h_{2}{|}^{2}{\left|h_{3}\right|}^{2}+C}\frac{E_{s}}{\sigma_{1}^{2}}}\right]\;\\ & =\int_{0}^{\infty }\int_{0}^{\infty }\int_{0}^{\infty }e^{-s\frac{xy}{y\left(1+z\frac{E_{i}}{\sigma_{1}^{2}}\right)+C}\frac{E_{s}}{\sigma_{1}^{2}}}f_{|h_{1}{|}^{2}}\left(x\right)f_{|h_{2}{|}^{2}}\left(y\right)f_{|h_{3}{|}^{2}}\left(z\right)dxdydz \end{array}$$

We define the following integral from the above triple-integral:

(10) $$I_{1}≜\int_{0}^{\infty }e^{-s\frac{xy}{y\left(1+z\frac{E_{i}}{\sigma_{1}^{2}}\right)+C}\frac{E_{s}}{\sigma_{1}^{2}}}f_{|h_{1}{|}^{2}}\left(x\right)dx$$

By using identities from [23] (Equations (7.621.4, 9.121.1)), we obtain the following form for $I_{1}$ as:

(11) $$\begin{array}{ccc} I_{1} & = & \left(\alpha_{1}\left(1+z\frac{E_{i}}{\sigma_{1}^{2}}\right)y+\alpha_{1}C\right){\left(\left(s\frac{E_{s}}{\sigma_{1}^{2}}+\beta_{1}\left(1+z\frac{E_{i}}{\sigma_{1}^{2}}\right)\right)y+C\beta_{1}\right)}^{m_{1}-1}\;\\ & & \times \;{\left(\left(s\frac{E_{s}}{\sigma_{1}^{2}}+\left(\beta_{1}-\delta_{1}\right)\left(1+z\frac{E_{i}}{\sigma_{1}^{2}}\right)\right)y+C\left(\beta_{1}-\delta_{1}\right)\right)}^{-m_{1}} \end{array}$$

Let us now define the following integral from Equation (9) by using $I_{1}$:

(12) $$I_{2}≜\int_{0}^{\infty }I_{1}f_{|h_{2}{|}^{2}}\left(y\right)dy$$

We substitute Equations (4) and (11) in Equation (12) and reach at:

(13) $$\begin{array}{ccc} I_{2} & = & \int_{0}^{\infty }\lambda_{2}y^{m_{2}-1}e^{-\epsilon_{2}y}\left(\alpha_{1}\left(1+z\frac{E_{i}}{\sigma_{1}^{2}}\right)y+\alpha_{1}C\right){\left(\left(s\frac{E_{s}}{\sigma_{1}^{2}}+\beta_{1}\left(1+z\frac{E_{i}}{\sigma_{1}^{2}}\right)\right)y+C\beta_{1}\right)}^{m_{1}-1}\;\\ & & \times \;{\left(\left(s\frac{E_{s}}{\sigma_{1}^{2}}+\left(\beta_{1}-\delta_{1}\right)\left(1+z\frac{E_{i}}{\sigma_{1}^{2}}\right)\right)y+C\left(\beta_{1}-\delta_{1}\right)\right)}^{-m_{1}}dy \end{array}$$

By employing the method outlined in [6], Equation (13) becomes:

(14) $$\begin{array}{ccc} I_{2} & = & \lambda_{2}\int_{0}^{\infty }\left(\alpha_{1}\left(1+z\frac{E_{i}}{\sigma_{1}^{2}}\right)y+\alpha_{1}C\right){\left(\left(s\frac{E_{s}}{\sigma_{1}^{2}}+\beta_{1}\left(1+z\frac{E_{i}}{\sigma_{1}^{2}}\right)\right)y+C\beta_{1}\right)}^{c_{1}}\;\\ & & \times \;{\left(\left(s\frac{E_{s}}{\sigma_{1}^{2}}+\left(\beta_{1}-\delta_{1}\right)\left(1+z\frac{E_{i}}{\sigma_{1}^{2}}\right)\right)y+C\left(\beta_{1}-\delta_{1}\right)\right)}^{-m_{1}}\;\\ & & \times \;{\left(\left(s\frac{E_{s}}{\sigma_{1}^{2}}+\beta_{1}\left(1+z\frac{E_{i}}{\sigma_{1}^{2}}\right)\right)y+C\beta_{1}\right)}^{e}y^{m_{2}-1}e^{-\epsilon_{2}y}dy \end{array}$$

where $c_{1}=\left\lfloor m_{1}\right\rfloor -1$ and $e=m_{1}-\left\lfloor m_{1}\right\rfloor$ for $m_{1}>1$; $c_{1}=0$ and $e=m_{1}-1$ for $m_{1}\leq 1$; and ⌊x⌋ denotes the largest integer not greater than *x*. By the use of binomial expansion in Equation (14), we rewrite Equation (14) as:

(15) I2=λ2∑l=0c1c1lsEsσ12+β11+zEiσ12lCβ1c1−l∫0∞α11+zEiσ12y+α1C×sEsσ12+β1−δ11+zEiσ12y+Cβ1−δ1−m1×sEsσ12+β11+zEiσ12y+Cβ1eym2+l−1e−ϵ2ydy

Now, we use the following approximation ${\left(1+x\right)}^{\eta }\approx 1+\eta x$, x<1 in Equation (15), and after some algebra involving series and integrals (for details, see Appendix I in [6]/Appendix II in [20]), we obtain:

(16) $$I_{2}≅P_{1}-P_{2}+P_{3}$$

where:

(17) P1=∑l=0c1c1lλ2Cβ1m1+m2−lsEsσ12+β11+zEiσ12m2∫011+zEiσ12α1Cβ1xsEsσ12+β11+zEiσ12+α1C×sEsσ12+β1−δ11+zEiσ12Cβ1xsEsσ12+β11+zEiσ12+C(β1−δ1)−m1×e−ϵ2Cβ1xsEsσ12+β11+zEiσ12xm2+l−1(1+ex)dx

(18) P2=∑l=0c1c1lλ2Cβ1m1+m2−lsEsσ12+β11+zEiσ12m2∫011+zEiσ12α1Cβ1xsEsσ12+β11+zEiσ12+α1C×sEsσ12+β1−δ11+zEiσ12Cβ1xsEsσ12+β11+zEiσ12+C(β1−δ1)−m1×e−ϵ2Cβ1xsEsσ12+β11+zEiσ12xe+m2+l−11+exdx

(19) P3=λ2∑l=0c1c1lsEsσ12+β11+zEiσ12l+eCβ1c1−l×α1f(e+m2+l)+α1C2β1esEsσ12+β11+zEiσ12f(e+m2+l−2)+α1C1+1+zEiσ12β1esEsσ12+β11+zEiσ12f(e+m2+l−1)

In Equation (19), f(.) can be evaluated as in the following equation:

$$f\left(a\right)=\frac{C^{a+1-m_{1}}{\left(\beta_{1}-\delta_{1}\right)}^{a+1-m_{1}}}{{\left(s\frac{E_{s}}{\sigma_{1}^{2}}+\left(\beta_{1}-\delta_{1}\right)\left(1+z\frac{E_{i}}{\sigma_{1}^{2}}\right)\right)}^{a+1}}\int_{0}^{\infty }e^{\frac{-\epsilon_{2}C\left(\beta_{1}-\delta_{1}\right)t}{s\frac{E_{s}}{\sigma_{1}^{2}}+\left(\beta_{1}-\delta_{1}\right)\left(1+z\frac{E_{i}}{\sigma_{1}^{2}}\right)}}t^{a}{\left(1+t\right)}^{-m_{1}}dt$$

The MGF of the considered network can now be written, by using Equations (5), (9) and (16), as:

(20) $$\begin{array}{ccc} M_{\gamma }\left(s\right) & ≜ & \int_{0}^{\infty }I_{2}f_{|h_{3}{|}^{2}}\left(z\right)dz\;\\ & = & \int_{0}^{\infty }I_{2}\lambda_{3}z^{m_{3}-1}e^{-\epsilon_{3}z}dz \end{array}$$

By putting Equation (16) in Equation (20), and after rearrangement of integrals, sums and manipulations, we finally write the MGF of our proposed system as:

(21) $$M_{\gamma }\left(s\right)=M_{1}-M_{2}+M_{3}+M_{4}+M_{5}$$

In Equation (21):

(22) M1=λ2λ3Cβ1m1+m2−1∑l=0c1c1l∫0∞zm3−1e−ϵ3zsEsσ12+β11+zEiσ12m2dz×∫01e−ϵ2Cβ1xsEsσ12+β11+zEiσ12xm2+l−1(1+ex)1+zEiσ12α1Cβ1xsEsσ12+β11+zEiσ12+α1C×sEsσ12+β1−δ11+zEiσ12Cβ1xsEsσ12+β11+zEiσ12+C(β1−δ1)−m1dx

(23) M2=λ2λ3Cβ1m1+m2−1∑l=0c1c1l∫0∞zm3−1e−ϵ3zsEsσ12+β11+zEiσ12m2dz×∫01e−ϵ2Cβ1xsEsσ12+β11+zEiσ12xe+m2+l−11+ex1+zEiσ12α1Cβ1xsEsσ12+β11+zEiσ12+α1C×sEsσ12+β1−δ11+zEiσ12Cβ1xsEsσ12+β11+zEiσ12+C(β1−δ1)−m1dx

(24) M3=α1λ2λ3C1+c1+e+m2−m1∑l=0c1c1lβ1c1−lβ1−δ1e+l+m2−m1×∫0∞1+1+zEiσ12β1esEsσ12+β11+zEiσ12sEsσ12+β11+zEiσ12e+l×sEsσ12+β1−δ11+zEiσ12−(e+l+m2)zm3−1e−ϵ3zdz×∫0∞e−ϵ2Cβ1tsEsσ12+β1−δ11+zEiσ12te+l+m2−11+t−m1dt

(25) M4=α1λ2λ3C1+c1+e+m2−m1∑l=0c1c1lβ1c1−lβ1−δ1e+l+m2+1−m1×∫0∞1+zEiσ12sEsσ12+β11+zEiσ12e+l×sEsσ12+β1−δ11+zEiσ12−(e+l+m2+1)zm3−1e−ϵ3zdz×∫0∞e−ϵ2Cβ1tsEsσ12+β1−δ11+zEiσ12te+l+m21+t−m1dt

(26) M5=α1λ2λ3C1+c1+e+m2−m1∑l=0c1c1lβ1c1−l+1β1−δ1e+l+m2−m1−1×∫0∞sEsσ12+β11+zEiσ12e+l−1×sEsσ12+β1−δ11+zEiσ12−(e+l+m2−1)zm3−1e−ϵ3zdz×∫0∞e−ϵ2Cβ1tsEsσ12+β1−δ11+zEiσ12te+l+m2−21+t−m1dt

It can be noticed from Equation (21) that MGF contains finite and infinite integrals, which can be accurately/easily calculated by using numerical computing packages, such as MATLAB or Mathematica.

### 3.2. Calculation of SER

The SER of the considered HSTS for M-PSK modulation is given by [22] as:

(27) $$P_{MPSK}=\frac{1}{\pi }\int_{0}^{\theta_{M}}M_{\gamma }\left(\frac{g_{MPSK}}{\sin^{2}\theta }\right)d\theta$$

where $\theta_{M}=\frac{\pi (M-1)}{M}$ and $g_{MPSK}=\sin^{2}\left(\frac{\pi }{M}\right)$. Alternatively, one can use the following accurate approximation of Equation (27) developed by [27]:

(28) $$P_{MPSK}=\sum_{p=1}^{3}b_{p}M_{\gamma }\left(a_{p}\right)$$

where $b_{1}=\frac{\theta_{M}}{2\pi }-\frac{1}{6}$, $b_{2}=\frac{1}{4}$, $b_{3}=\frac{\theta_{M}}{2\pi }-\frac{1}{4}$, $a_{1}=g_{MPSK}$, $a_{2}=\frac{4}{3}g_{MPSK}$ and $a_{3}=\frac{g_{MPSK}}{\sin^{2}\left(\theta_{M}\right)}$. The approximate average SER of the considered HSTS is finally obtained from Equations (21) and (28). Similarly to M-PSK, the approximate average SER of M-PAM signal can be calculated as:

$$P_{MPAM}=\left(1-\frac{1}{M}\right)\left(\frac{1}{6}M_{\gamma }\left(\frac{3}{M^{2}-1}\right)+\frac{1}{2}M_{\gamma }\left(\frac{4}{M^{2}-1}\right)\right)$$

Similarly to M-PSK, the approximate average SER of the M-QAM signal can be calculated as:

$$\begin{array}{ccc} P_{MQAM} & = & \left(1-\frac{1}{\sqrt{M}}\right)\left(\frac{1}{3}M_{\gamma }\left(\frac{3}{M-1}\right)+M_{\gamma }\left(\frac{4}{M-1}\right)\right)\\ & & -\frac{1}{6}{\left(1-\frac{1}{\sqrt{M}}\right)}^{2}\left(3M_{\gamma }\left(\frac{6}{M-1}\right)+M_{\gamma }\left(\frac{3}{M-1}\right)\right) \end{array}$$

## 4. Asymptotic SER

In this section, we present an accurate approximation for the average SER, which provides a reasonable insight into the performance of the system under consideration in the high SNR regime. Let us assume that when SNR takes a very large value, then $m_{1}$ is approximated by its closest integer [6,21]; therefore, by using the Binomial expansion in Equation (11), we have:

(29) I1=∑l=0m1−1m1−1lα11+zEiσ12y+α1CsEsσ12+β11+zEiσ12lylCβ1m1−l−1sEsσ12+β1−δ11+zEiσ12y+Cβ1−δ1m1

By substituting Equation (4) in Equation (12), we get:

(30) $$I_{2}=\int_{0}^{\infty }I_{1}\lambda_{2}y^{m_{2}-1}e^{-\epsilon_{2}y}dy$$

By substituting Equation (29) in Equation (30) and after interchanging summation and integration, we get:

(31) I2=λ2∑l=0m1−1m1−1lsEsσ12+β11+zEiσ12lCβ1m1−l−1sEsσ12+β1−δ11+zEiσ12m1∫0∞α11+zEiσ12y+α1Cym2+l−1y+Cβ1−δ1sEsσ12+β1−δ11+zEiσ12m1eϵ2ydy

After multiplication and the simplification of terms inside the integral in Equation (31), we obtain:

(32) I2=λ2∑l=0m1−1m1−1lsEsσ12+β11+zEiσ12lCβ1m1−l−1sEsσ12+β1−δ11+zEiσ12m1×α11+zEiσ12∫0∞ym2+le−ϵ2yy+Cβ1−δ1sEsσ12+β1−δ11+zEiσ12m1dy+α1C∫0∞ym2+l−1e−ϵ2yy+Cβ1−δ1sEsσ12+β1−δ11+zEiσ12m1dy

By using [28] (Equation (2.3.6.9)), we can solve the integrals in Equation (32) and get:

(33) I2=λ2∑l=0m1−1m1−1lsEsσ12+β11+zEiσ12lCβ1m1−l−1sEsσ12+β1−δ11+zEiσ12m1×α1Γ(m2+l+1)1+zEiσ12Cβ1−δ1sEsσ12+β1−δ11+zEiσ12m2+l+1−m1×Ψm2+l+1,m2+l+2−m1;ϵ2Cβ1−δ1sEsσ12+β1−δ11+zEiσ12+α1CΓ(m2+l)Cβ1−δ1sEsσ12+β1−δ11+zEiσ12m2+l−m1×Ψm2+l,m2+l+1−m1;ϵ2Cβ1−δ1sEsσ12+β1−δ11+zEiσ12

where Ψ(a,b;z) is defined in [23] (Equation (9.210.2)). By substituting Equations (5) and (33) in Equation (20), using the fact that $\bar{\gamma }$ takes very large value and employing [23] (Equation (9.211.4)), we finally get the MGF for the high SNR case as:

(34) $$M_{\gamma }\left(s\right)≅T_{1}+T_{2}$$

where:

(35) T1=α1λ2λ3Cm2∑l=0m1−1m1−1lβ1m−l−1β1−δ1m2+l+1−m1×∫0∞1+zEiσ12sEsσ12+β11+zEiσ12lsEsσ12+β1−δ11+zEiσ12−(m2+l+1)zm3−1e−ϵ3zdz×∫0∞e−ϵ2Cβ1tsEsσ12+β1−δ11+zEiσ12tm2+l1+t−m1dt

and:

(36) T2=α1λ2λ3Cm2∑l=0m1−1m1−1lβ1m−l−1β1−δ1m2+l−m1×∫0∞sEsσ12+β11+zEiσ12lsEsσ12+β1−δ11+zEiσ12−(m2+l)zm3−1e−ϵ3zdz×∫0∞e−ϵ2Cβ1tsEsσ12+β1−δ11+zEiσ12tm2+l−11+t−m1dt

By considering the terms corresponding to $l=m_{1}-1$ in Equation (34), the asymptotic average SER of the considered HSTS employing M-PSK/M-QAM/M-PAM, can finally be obtained from Equation (34) and expressions for $P_{MPSK}/P_{MQAM}/P_{MPAM}$ in Section 3.2.

## 5. Numerical Results

This section presents the analytical and simulated results of the considered HSTS model using M-PSK, M-QAM and M-PAM modulation formats over generalized fading channels. We demonstrate the usefulness of the expressions derived in Section 3 and Section 4 using numerical examples and study the effects of interference on the system’s performance. The simulated results are obtained by generating $10^{7}$ channel realizations for the M-PSK, M-QAM and M-PAM symbols. It is assumed that relay-destination and interferer-relay channels follow the Nakagami-*m* fading with parameters adopted from [29]. The satellite-relay LMS channel is changed according to different practical shadowing conditions. The parameters of the shadowed Rice LMS model are shown in Table 1; see Section 2 for more details on these channel parameters.

**Figure 2:**
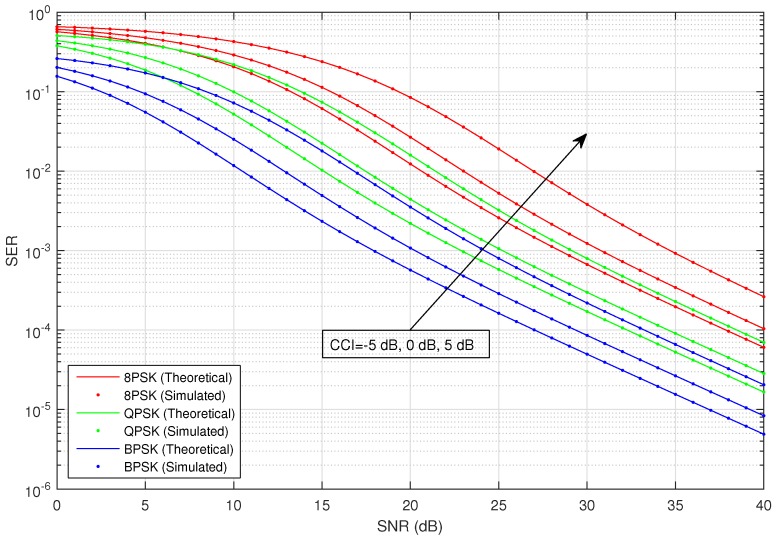
Average symbol error rate (SER) versus SNR of M-PSK with varying CCI and the LMS channel in infrequent light shadowing.

**Figure 3:**
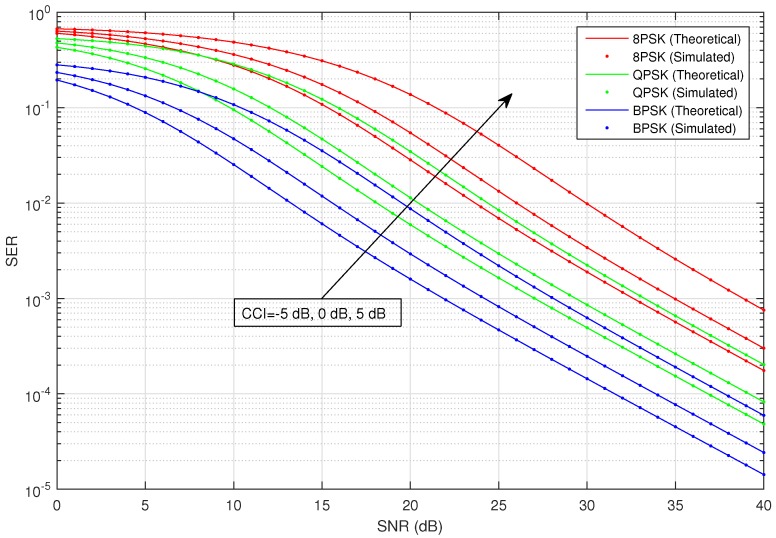
Average SER versus SNR of M-PSK with varying CCI and the LMS channel in average shadowing.

**Figure 4:**
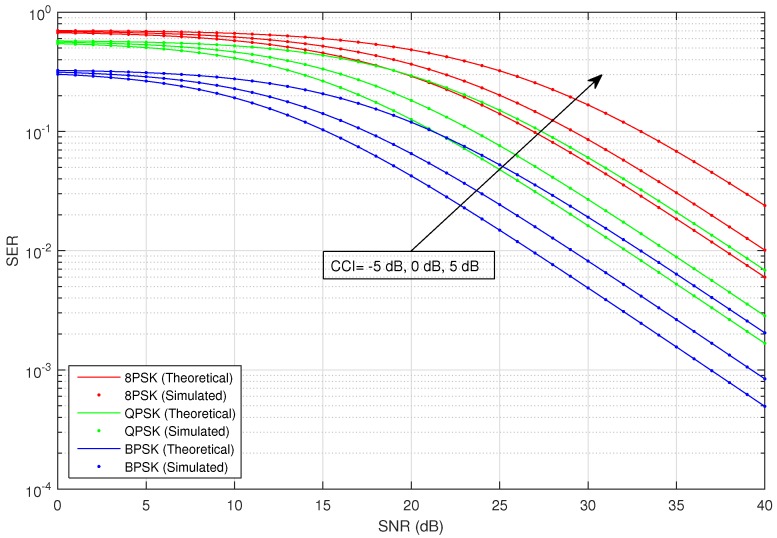
Average SER versus SNR of M-PSK with varying CCI and the LMS channel in frequent heavy shadowing.

**Figure 5:**
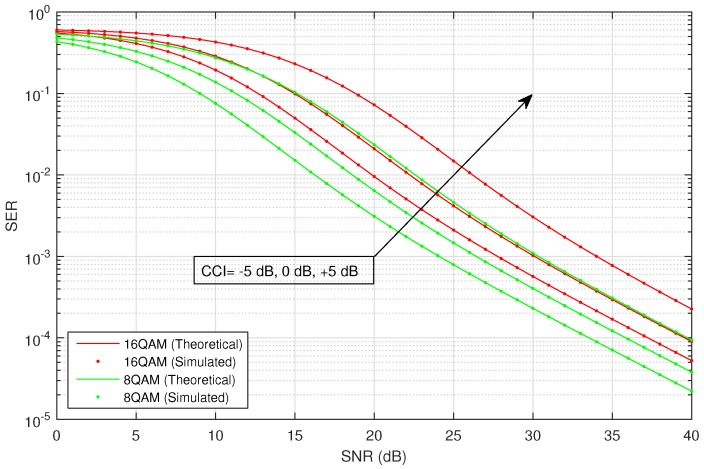
Average SER versus SNR for M-QAM with CCI in infrequent light shadowing.

**Figure 6:**
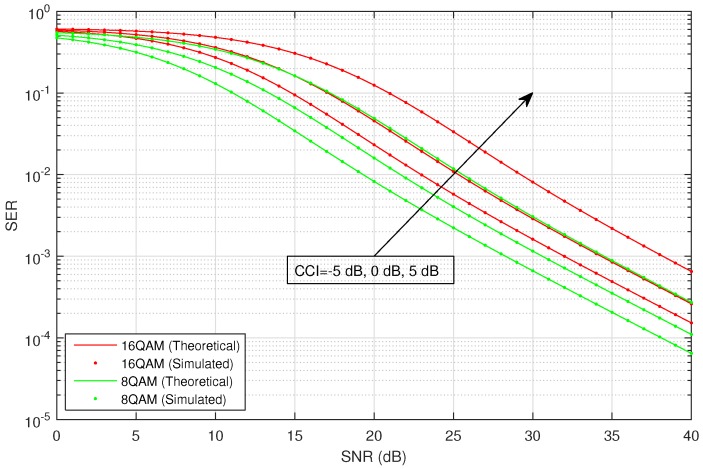
Average SER versus SNR for M-QAM with CCI in average shadowing.

**Figure 7:**
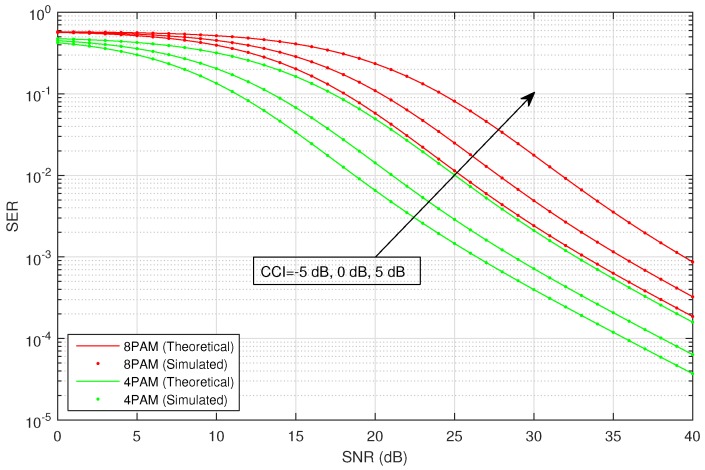
Average SER versus SNR for M-PAM with CCI in infrequent light shadowing.

Figure 2 shows the average SER versus SNR of the considered HSTS, for infrequent light shadowing (in satellite-relay LMS channel), with multiple values of CCI (−5 dB, 0 dB and 5 dB) using different M-PSK modulation schemes: BPSK, QPSK and 8-PSK. It is assumed that $\sigma_{1}^{2}=\sigma_{2}^{2}$; and on the x-axis of Figure 2, SNR denotes $\bar{\gamma }$; in the plots, CCI represents ${\bar{\gamma }}_{int}$. We consider the situation when relay is interfered by a single dominant interferer; we assume that the interferer can operate in a range of different transmit power levels. The value of total CCI ${\bar{\gamma }}_{int}$ experienced by the source to relay link is varied in increasing order as: −5 dB, 0 dB and 5 dB. An arrow shown in Figure 2 traversing the plots represents the direction of increasing CCI, i.e., from −5 dB to +5 dB. The analytical average SER for M-PSK is plotted by using Equation (28), and that for M-QAM and M-PAM is evaluated by employing the corresponding expressions given in Section 3. Figure 3 shows the average SER versus SNR of the considered HSTS, for average shadowing (in satellite-relay LMS channel), with multiple values of CCI (−5 dB, 0 dB and 5 dB) using different M-PSK modulation schemes: BPSK, QPSK and 8-PSK. Figure 4 shows the average SER versus SNR of the considered HSTS, for frequent heavy shadowing (satellite-relay LMS channel), with multiple values of CCI (−5 dB, 0 dB and 5 dB) using different M-PSK modulation schemes: BPSK, QPSK and 8-PSK. Figure 5 shows the average SER versus SNR of the considered HSTS, for infrequent light shadowing (in satellite-relay LMS channel), with multiple values of CCI (−5 dB, 0 dB and 5 dB) using different M-QAM modulation schemes: 8-QAM and 16-QAM. Figure 6 shows the average SER versus SNR of the considered HSTS, for average shadowing (satellite-relay LMS channel), with multiple values of CCI (−5 dB, 0 dB and 5 dB) using different M-QAM modulation schemes: 8-QAM and 16-QAM. Figure 7 shows the average SER versus SNR of the considered HSTS, for infrequent light shadowing (in satellite-relay LMS channel), with multiple values of CCI (−5 dB, 0 dB and 5 dB) using different M-PAM modulation schemes: 4-PAM and 8-PAM. The above discussion about the values of network and interference parameters for Figure 2 is also applicable for Figure 3, Figure 4, Figure 5, Figure 6 and Figure 7. We observe from Figure 3, Figure 4, Figure 5, Figure 6 and Figure 7 that the simulated SER very closely follows the analytical SER for all shadowing conditions of the LMS channel and modulations considered in the figures; indicating the correctness of the approximations taken in deriving the performance analysis framework of Section 3.

As we can notice from Figure 2, Figure 3, Figure 4, Figure 5, Figure 6 and Figure 7, when CCI at the relay increases from −5 dB to +5 dB, there is clearly a notable increase in average SER of the considered HSTS for a given modulation scheme. We see that the increase in SER is more prominent for higher-order modulation, such as 8-PSK/16-QAM, than that for the lower-order modulation scheme of BPSK/8-QAM/4-PAM. This can be seen from Figure 2, Figure 3 and Figure 4, e.g., by comparing the plots for 8-PSK and BPSK for the same value of given CCI. The same line of reasoning is also applicable for comparing combinations of 8-PSK/QPSK and QPSK/BPSK modulations. When source-relay LMS channel suffers an increase in the amount of shadowing, as shown by the sequence of Figure 2, Figure 3, Figure 4, Figure 5, Figure 6 and Figure 7, respectively, we notice that the average SER of the HSTS also increases correspondingly. The reader can view the effect of shadowing (in the source-relay LMS channel) on the considered system by comparing the curves for particular modulation (M-PSK) with the same given CCI from Figure 2, Figure 3 and Figure 4, and also, the same observation is valid for M-QAM from Figure 5 and Figure 6. We also comment here about the computational complexity of the expression for average SER in Equation (28), since it contains multiple integrals. We tested in MATLAB that for different SNRs (dB), e.g., 10, 20, 30; a modern personal computer takes approximately 0.1 s to calculate Equation (28).

**Figure 8:**
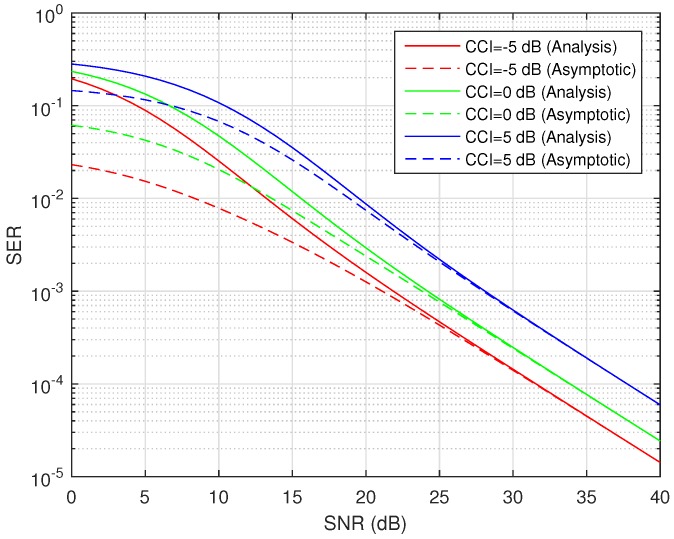
Average SER/asymptotic SER versus SNR for BPSK with CCI in average shadowing.

**Figure 9:**
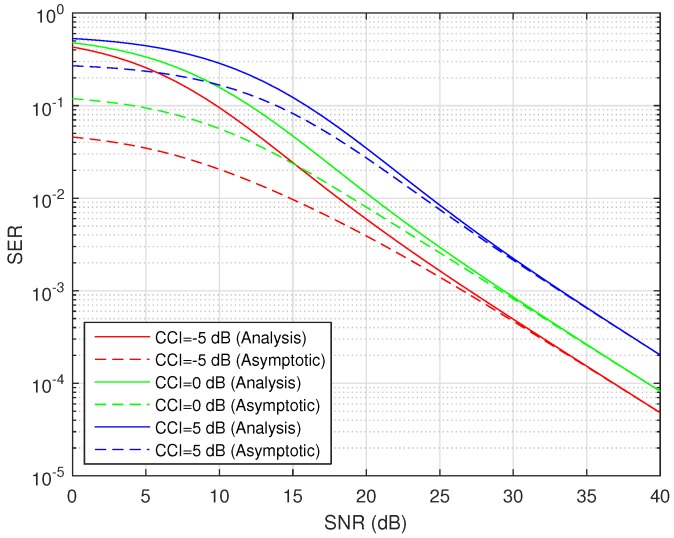
Average SER/asymptotic SER versus SNR for QPSK with CCI in average shadowing.

The difference between exact average SER and asymptotic average SER (high SNR approximation) is plotted in Figure 8. The analysis is performed for BPSK-modulated HSTS with different values of CCI over the average shadowed LMS source-relay channel. It can be seen clearly from Figure 8 that with the increasing value of SNR, the gap between the exact SER curve and the asymptotic SER curve keeps on decreasing. The difference between the exact and asymptotic values is very small in the high SNR region of 25–30 dB, and when SNR is over 30 dB, the magnitude of the difference between exact and asymptotic values is unnoticeable. Another example of the difference between exact average SER and asymptotic average SER (high SNR approximation) is presented in Figure 9. The analysis is done for QPSK-modulated HSTS with different values of CCI over average shadowed LMS source-relay channel. It can be noticed clearly from Figure 9 that with the increasing value of SNR, the gap between the exact SER curve and asymptotic SER curve keeps on decreasing. The difference between the exact and asymptotic values is very small in the high SNR region of 25–30 dB, and when SNR is over 30 dB, the magnitude of the difference between exact and asymptotic values is unnoticeable. As discussed above, Figure 8 and Figure 9 indicate the correctness of the assumptions and approximations taken for deriving the asymptotic SER. The so-derived approximation is a useful tool for substituting the computationally-intricate formulation inherent to the exact expression of Equation (28).

## 6. Conclusions

In this paper, we studied the SER performance of AF-based HSTS with a dominant co-channel interferer at the relay. The performance analysis framework was developed by deriving new analytical expressions for the average SER of commonly-used modulation techniques: M-PSK, M-QAM and M-PAM. Simple asymptotic expressions for the corresponding exact analytical expressions were also presented. The presented analyses showed that CCI causes significant degradation in the SER performance of the considered HSTS. Our comprehensive analysis was proven to be useful in understanding how interference characteristics at the relay can degrade the overall system performance, depending on different system and network parameters. Future research may include different channel models for the satellite-terrestrial link, the effects of the fading parameters of the network channels and the performance of other forwarding schemes at the relay.

## Figures and Tables

**Table 1:** LMS channel parameters [21].

Shadowing	*b*${}_{i}$	*m*${}_{i}$	Ω${}_{i}$
Frequent heavy	0.063	0.739	$8.97\times 10^{-4}$
Average	0.126	10.1	0.835
Infrequent light	0.158	19.4	1.29
